# Effect of guided counseling on nutritional status of pregnant women in West Gojjam zone, Ethiopia: a cluster-randomized controlled trial

**DOI:** 10.1186/s12937-020-00536-w

**Published:** 2020-04-28

**Authors:** Yeshalem Mulugeta Demilew, Getu Degu Alene, Tefera Belachew

**Affiliations:** 1 School of Public Health, College of Medicine and Health Sciences, Bahir Dar University, P.O. Box 79, Bahir Dar, Ethiopia; 2 Department of Nutrition and Dietetics, Faculty of Public Health, Jimma University, P.O. Box 378, Jimma, Ethiopia

**Keywords:** Nutritional status, Guided counseling, Intervention, Pregnant women

## Abstract

**Background:**

Undernutrition during pregnancy affects birth outcomes adversely. In Ethiopia, despite nutrition counseling on the maternal diet being given by the health workers during pregnancy, maternal undernutrition is still high in the country. Hence, this study aimed to assess the effect of guided counseling using the health belief model and the theory of planned behavior on the nutritional status of pregnant women.

**Methods:**

A two-arm parallel cluster randomized controlled community trial was conducted in West Gojjam Zone, Ethiopia, from May 1, 2018, to April 30, 2019. The nutritional status of the women was assessed using mid-upper arm circumference. A total of 694 pregnant women were recruited from the intervention (n=346 ) and control (n=348) clusters. Of which endline data were collected from 313 and 332 pregnant women in the intervention and control clusters, respectively. The intervention was started before 16 weeks of gestation and pregnant women in the intervention group attended 4 counseling sessions. Counseling was given at the participants’ homes using a counseling guide with the core contents of the intervention. Leaflets with appropriate pictures and the core messages were given for women in the intervention arm.

Women in the control group got the routine nutrition education given by the health care system. Data were collected using interviewer administered structured questionnaires and mid-upper arm circumference was measured using an adult MUAC tape. Descriptive statistics  and linear mixed-effects model were used to assess the intervention effect after adjusting for potential confounders.

**Results:**

After the intervention, the prevalence of undernutrition was 16.7% lower in the intervention group compared with the control arm (30.6% Vs 47.3%, *P* = < 0.001). Women in the intervention group showed significant improvement in nutritional status at the end of the trial than the control group (β = 0.615, *p* = < 0.001).

**Conclusion:**

This study demonstrated that guided counseling using the health belief model and the theory of planned behavior was effective in improving the nutritional status of pregnant women. The results imply the need for the design of model and theory based nutrition counseling guidelines. The trial was registered in Clinical Trials.gov (NCT03627156).

## Introduction

Maternal nutrition during pregnancy shapes intrauterine programming, fetal growth, and development [1, 2]. It also determines child survival, the risk of developing chronic disease and human capital acquisition later in life [3]. Despite this, maternal malnutrition is high throughout the world, particularly in Asian and Sub-Saharan African countries [4, 5].

The prevalence of maternal undernutrition is persistently high in Ethiopia. According to the 2016 Ethiopian demographic and health survey (EDHS) report, nearly a quarter (22%) of reproductive age women were undernourished, while overweight or obese women constituted 8% [6]. According to Kedir H et al., (2016), one in four (24%) pregnant women were undernourished [7] showing that the prevalence of chronic energy deficiency was higher among Ethiopian pregnant women. There is such a pattern that undernutrition was higher among rural residents compared to urban dwellers (24.7% Vs 14.8%), while overweight or obesity was higher among urban dwellers (21.4%) than women who reside in rural areas (3.5%) [6].

Maternal undernutrition during pregnancy was associated with all causes of maternal mortality up to 42 days post-delivery [8]. Moreover, it affects short-and long-term health outcomes of the women and the growing fetus [9]. It was the underlying cause for the death of more than 3.5 million women and under 5 year old children. Additionally, poor dietary intake during the first thousand days of life causes permanent disability of millions more by affecting physical growth and mental development [8].

In Ethiopia, maternal and child mortality is still high, 412 maternal deaths per 100,000 live births and 67 child deaths per 1000 live births were reported in 2016 EDHS [6]. According to Yohannes T et al., (2017) [10]*,* among the common causes of child mortality, malnutrition was the underlying cause for 60.7, 52.3, 44.8, and 57.3% of deaths from diarrhea, pneumonia, measles, and malaria, respectively. On the other hand, maternal obesity and excessive gestational weight gain could increase the risk of fetal congenital anomalies and obstetric complications [8].

The nutritional status of pregnant women can be assessed using body mass index and mid-upper arm circumference (MUAC) measurement [11]. But, pregnancy-related weight gain affects the reliability of using body mass index to assess the nutritional status of pregnant women. Taking this into consideration, in this study MUAC was used to determine the nutritional status of the women [12, 13].

Nutrition education interventions were effective in improving the gestational weight gain and nutritional status of pregnant women [14, 15]. Overall, improvements in women’s nutritional status are associated with positive effects on pregnancy outcomes and child survival [16]. Moreover, nutrition education interventions that improve maternal nutritional status are among the most effective strategies in promoting maternal and child health [16].

However, in Ethiopia, the routine nutrition education given by the health system is “vague and inconsistent.” Professionals have been advising pregnant women to eat one additional meal from available foodstuffs [17]. As a result, maternal and child undernutrition remains a major public health problem in the country [6]. Therefore, appropriate counseling on the maternal diet during pregnancy seems to be of high priority to promote positive pregnancy outcomes [18].

Selecting an appropriate model for counseling pregnant women is the first step during planning nutrition education to bring positive results. Besides, from literature, nutrition counseling interventions using the Health belief model (HBM) or Theory of planned behavior (TPB) are effective to improve maternal knowledge on diet during pregnancy, dietary practice, nutritional status and birth outcomes [19, 20]. Moreover, evidence support the use of multiple behavioral theories during counseling since a single theory does not fully explain dietary behavior [20, 21]. Thus, this study used the HBM and TPB during counseling.

Counseling is a series of professional guidance that aims to change the knowledge, attitude, and behavior of an individual [22]. In this study, guided counseling refers to professional guidance based on the HBM and TPB to encourage positive behavior outcomes [18, 23]. The HBM contains numerous principal concepts that predict the reason why people take measures to prevent illness. These constructs of HBM are perceived susceptibility, perceived seriousness, perceived benefits and perceived barriers to a behavior, cues to action, and self-efficacy [18, 23].

According to the TPB, the intention is the direct determinant of behavior. The intention, in turn, is influenced by a person’s attitude towards a given behavior, an individual’s perception of social pressures caused by important people in practicing or not practicing a specific behavior, and perceived behavioral control [24].

In this intervention, each woman attended four counseling sessions. Since the nutrient requirement increases with increasing gestational age, the women were counseled to increase portion size and frequency of meal with increasing gestational age. Counseling was also given on improving the diversity of meals, taking iron/folic acid supplement and using health services. Moreover, leaflets with core messages and appropriate pictures were given to the women to use it at home. The intervention was described in detail in the methods part.

In Ethiopia, data on the effect of nutrition education intervention on the nutritional status of pregnant women were scarce. Thus, the objective of this study was to assess the effect of guided counseling using the health belief model (HBM) and the theory of planned behavior (TPB) in improving the nutritional status of pregnant women in West Gojjam Zone. The results of the study could be an input to policymakers and planners at the national and regional level to amend nutrition counseling methods.

## Methods and materials

### Study design, setting and ethics

This study was a 1 year two-arm parallel design cluster randomized controlled community trial. Clusters (kebeles or the smallest administrative units in Ethiopia) were taken as a unit of randomization. The study was conducted in West Gojjam Zone from May 1, 2018, to April 30, 2019. It is one of the 11 zones in *Amhara* Region comprised of 15 *woredas*/districts/ with a total population of 2,641,240, half of which (50.7%) were females [25]. The number of estimated pregnant women was 61,072.

**Fig. 1:**
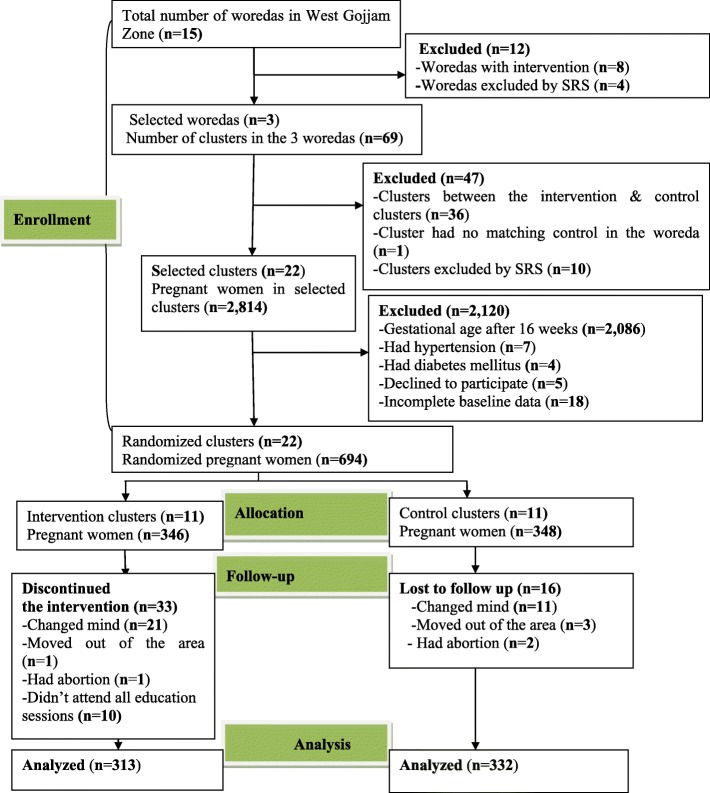
This figure shows the flow of the study participants through the trial according to the criteria recommended in the CONSORT guideline

The study was conducted in accordance with the principles of Helsinki Declaration and the requirements of Good Clinical Practice [26]. The research protocol was approved by the Institutional Review Board of Bahir Dar University (protocol number: 092/18–04). Written informed consent (fingerprint for women who could not read and write) was secured from each participant prior to starting the trial. The trial was registered in the Clinical Trials.gov (NCT03627156). Consolidated Standards of Reporting Trials (CONSORT) guideline was used for reporting the results (Fig. 1 and Additional file 1) [27].

### Participants and sample size determination

The study targeted pregnant women before 16 weeks of gestation. Women who had no intention of leaving the study area until delivery were included in this study. Women with confirmed or diagnosed hypertension and/or diabetes mellitus were excluded from participating in the study [28]. The sample size was calculated using G power 3.1.9.2 program with a power of 85% for Fisher’s exact test and precision of 5%. According to Kedir H et al., (2016) the prevalence of undernutrition among pregnant women (p1) was 24% [7] and P2 was 9% by assuming a 15% difference between p1 and P2 [29].

The calculated sample size was multiplied by design effect of two due to cluster sampling. Considering a 10% loss to follow up, the final sample size was **214** pregnant women in each arm. Since cluster randomization was used and pregnant women who fulfilled the requirement were included, **346** women in the intervention group and **348** women in the control group were enrolled in this study.

### Recruitment, randomization and intervention allocation

From 15 *woredas* in the zone, eight had nutrition education intervention on complementary feeding practice. These eight woredas were excluded from the study. From the seven eligible *woredas,* three *woredas* namely: *Bahir Dar Zuria Woreda, South Achefer Woreda,* and *Burie Zuria Woreda* were selected using simple random sampling (SRS) technique. Then, samples of non-adjacent clusters were selected from the three woredas using SRS (lottery) method. The following formula was used to determine the number of clusters [30].

$$ C=\frac{1+{\left( Z\alpha /{}_2+{Z}_{\beta}\right)}^2\left[\frac{P_0\left(1-{P}_0\right)}{n}+\frac{P_1\left(1-{P}_1\right)}{n}+{K}^2\left({P}_0^2+{P}_1^2\right)\right]}{{\left({P}_0-{P}_1\right)}^2} $$

Where c is the number of required clusters, Po was undernourished pregnant women (24%) [7], p_1_ was the expected number of undernourished women after intervention (9%) by assuming 15% difference between p_0_ and p_1,_ n was the number of households that had pregnant women in each cluster (assuming an average of 35 women). K was a coefficient of variation of undernourished women between clusters within each arm. Since there was no study to estimate K, it was taken as 0.5. Therefore, 11 clusters per arm were included in this study**.**

Based on proportional to size allocation**,** ten clusters from Bahir Dar Zuria Woreda, six clusters from South Achefer Woreda and another six clusters from Burie Zuria Woreda were selected, randomly. Finally, SRS (lottery) method and a 1:1 ratio were used to allocate intervention and control clusters (Additional file 2).

Cluster randomization was used to prevent message contamination because women in the same cluster had a high probability of communicating and discussing the intervention messages. To avoid this, all eligible pregnant women in one cluster were enrolled in the same arm (either in the intervention or control arms). Moreover, buffer zones (non-selected clusters) were also left between the intervention and control clusters to prevent information contamination [31].

Eligible pregnant women were screened through the house to house survey by inquiring about the first date of the last menstrual period and confirming pregnancy with a pregnancy test. All eligible pregnant women were included in the study. Nurses working in selected woredas randomized the cluster, screened and enrolled the study participants from May to August 2018.

### Intervention

Community-based guided counseling using the HBM and the TPB was the intervention package for this study (Additional file 3). It was adapted from the recommendations by World Health Organization and Ministry of Health of Ethiopia [32, 33]. The core contents of the counseling guide were increasing meal frequency and portion size with increasing gestational age. Message on taking diversified meals by giving emphasis to iron-rich foods, animal products, fruits, and vegetables was also one component of the counseling guide. Messages on the consumption of iron/folic acid supplement and iodized salt were also included in the core contents of the counseling guide. Additional messages of the core contents were reducing heavy workload, taking day rest, impregnated bed net use and utilization of health care services.

Moreover, the consequences of taking inadequate nutrient, susceptibility to and severity of the consequences of insufficient nutrient intakes were also discussed during counseling. The benefits of taking an adequate amount of diversified meals and barriers that interfere with taking a balanced diet were also included in the counseling guide. Attitude, subjective norms, self-efficacy, perceived control, intention, knowledge and dietary practice were assessed during each counseling session. Then, counseling was given based on the identified gaps and household income.

Each pregnant woman attended four counseling sessions throughout her entire pregnancy. Individual nutrition counseling was given through a home visit on non-working days (religious holidays and weekends). During counseling, counselors used a client-centered approach to identify women’s dietary practices and their specific needs in terms of nutrition. Counselors considered women’s needs, household income and identified gaps and allowed the women to choose recommendations that were locally available, acceptable and affordable.

Counseling was delivered monthly using a counseling guide with the core contents and each counseling session lasted for 40 to 60 minutes. The first counseling was given before 16 weeks of gestation, focused on basic nutrition, food groups, food selection, preparation, meal frequency, portion size, and iodized salt utilization. The second and third sessions of the counseling were given during the second trimester of pregnancy and covered the whole contents of the counseling guide. The last counseling was given based on the identified gaps during the early third trimester of pregnancy.

Leaflets with the core messages in Amharic (local language) and appropriate pictures were prepared and delivered to each pregnant woman in the intervention arm. For women who couldn’t read, anyone at home or in the neighborhood who could read was requested to read the leaflet to the woman and other family members.

Women in the control arm received nutrition education given by the health care system. Pregnant women from both the control and intervention arms had access to ANC services. Six BSc nurses and three MSc nutritionists were recruited as counselors and supervisors of the counseling process, respectively. Counselors were selected based on their previous experience in giving counseling services. A three-day intensive training with role-playing and fieldwork were given to the counselors and supervisors using the training manual. Moreover, a one-day additional training was given for the counselors and supervisors after two months of intervention implementation to keep providers sticking to the standardized procedures over time.

### Intervention fidelity

Criteria were established to assess fidelity of the intervention, based on the National Institutes of Health Behavioural Change Consortium developed best practice recommendations [34]. The criteria included checklists to assess intervention design, training of counselors, counseling process, receipt of intervention and enactment of skills gained from the intervention [35].

The intervention design had theoretical ground. Non-adjacent clusters were selected to prevent information contamination. Equal numbers of clusters for the intervention and control groups were taken from each woreda to balance variations. The trial used a control group and counseling guide. The intervention process was pretested before the implementation of the trial. Besides, each woman received equal numbers and frequencies of counseling, and the lengths of contacts within an intervention group were similar to make the process standardized.

Counselor training was given in a group using a training manual, role-playing, and mock counseling practice. Counselors’ knowledge and skill were assessed by pre and post-training tests and practical evaluation. Counseling sessions were randomly selected for process evaluation and all selected sessions were evaluated by one process evaluator. The process observer rated the educator using a ‘yes/no’ rating system on items such as using a counseling guide, provision of the whole content, duration and frequency of counseling, preparedness, accuracy, and ability to properly respond to questions.

Intervention receipt was assessed using checklists on knowledge of the women on diet during pregnancy through interviewing about their understanding of the core contents of the intervention. Intervention enactment was also assessed using the checklist on a demonstration of food preparation and consumption.

Even if, participant allocation concealment was not possible due to the nature of the intervention, participants, counselors, and data collectors were blinded to the study hypotheses. Additionally, the data entry clerk was blinded by labeling the groups with a non-identifiable unique number until analysis was finalized. The counseling process was supervised by the counseling supervisors and principal investigator.

### Data collection and measurement

Six nurses collected data using structured questionnaires through one-to-one interview of the participants at their homes. The questionnaire included socio-demographic variables, obstetric history, HBM, and TPB constructs. Data on socio-demographic and obstetric characteristics were collected at the baseline. Whereas, data on food security, MUAC, HBM, and TPB constructs were taken before and after implementation of the intervention. Data collectors and supervisors were trained for 3 days using a training manual focused on the data collection tools, procedures, and ethical issues.

To prevent the breaching of privacy of the women, no one was allowed to have free access to the place where the interview was conducted. The supervisors and the principal investigator overhauled the data collection procedure. The data collection team held a daily meeting to discuss challenges encountered during the day. Besides, nutrition counselors and their supervisors also held a monthly meeting to discuss difficulties during nutrition counseling and feedback was given to the counselors.

The secondary outcome of this trial was the nutritional status of the pregnant women that was assessed by measuring MUAC. Post-intervention data were measured from 36 to 37 weeks of pregnancy. Women who didnot attend all counseling sessions were considered as ‘did not adhere- to the guideline’ and those withdraw from participating in the study were taken as ‘lost to follow up’.

### Mid upper arm circumference measurements

There is minimal change in MUAC during pregnancy, accordingly, MUAC is a better indicator of pre-pregnancy body fat and the nutritional status of pregnant women than body mass index [12, 36, 37]. Therefore, in this study, MUAC was used to assess the nutritional status of pregnant women and was measured on the upper left arm. During the procedure, the midpoint of the upper arm was located by flexing the women’s elbows to 90^0^ with the palm facing upwards. Then the distance from the acromion to olecranon processes was measured and the midpoint was marked. Finally, measuring tape was placed snugly around the arm at the midpoint mark while hanging arm freely, palm facing towards the thigh. Two measurements were taken and read the measurement to the nearest 0.1 cm. Women with MUAC > = 23 cm were considered normal nourished whereas participants with MUAC < 23 cm were labeled as undernourished [12, 13, 38].

The wealth index of the household was determined using principal component analysis (PCA) by considering latrine, water source, household assets, livestock, and agricultural land ownership. The responses of all non-dummy variables were classified into three parts, and the highest one was coded as 1 and the two lower values were given code 0. Factor scores were produced using variables having a commonality value of greater than 0.5 in PCA. Quintiles of the wealth score were created using the first principal component.

Food security status was assessed using 27 previously validated questions [39]. A household that experienced less than the first 2, 2–10, 11–17 and > 17 food insecurity indicators were considered as food secure, mildly, moderately and severely food insecure households, respectively. The attitude, knowledge, subjective norms, intention, perceived susceptibility, severity, benefit, and barriers were assessed using the sum of their respective composite questions. The full description of data collation, measurements, the study area and participants described elsewhere [28].

### Data management and analysis

Descriptive statistics were used to summarize the baseline socio-demographic characteristics of the women by group status. A chi-square test was performed to compare the baseline characteristics of the intervention and control groups. Comparisons of MUAC between and within the intervention and control groups were done using independent samples and paired sample t-tests, respectively.

A per-protocol analysis was performed in this study. The per-protocol analysis includes all the study participants who adhered to the predetermined guideline. Therefore, in this study, women who attended four education sessions and gave endline data were included in the analysis.

A linear mixed-effects model was used to determine the impacts of the intervention on changes in the nutritional status of pregnant women over time. This model enables to accommodate the correlation of observations due to the repeated measures (pre- and post-intervention) and the clustering of individuals within the 22 randomly selected clusters. During fitting the model, participants and clusters were analyzed as random effects. This model also enables to control the effects of potential confounding factors (food security, latrine utilization, education, family size, source of drinking water and age).

The intercept-only model estimates the variance of the cluster-level residual errors as 0.0035 (variability of the average nutritional status across all clusters was 0.0035 and which wasn’t statistically significant (*p* = 0.90). The intra-cluster correlation coefficient was closer to zero (0.001) which showed that no need for fitting a third-level model.

Therefore, the two-level model was fitted to account for time-invariant variables at the individual level. The effect of the intervention was evaluated by testing the interaction term between time and treatment allocation. All statistical analyses were performed using the SPSS package version 23.

## Results

### Socio-demographic characteristics of pregnant women

From 694 pregnant women who enrolled in this study, 645 **(IG = 313, CG = 332)** of them strictly adhered to the protocol and were included in the analysis. At the baseline, there was no significant difference in all socio-demographic characteristics between the intervention and control groups (*P* > 0.05). Table 1 presents the baseline characteristics of pregnant women.

**Table 1:** Socio-demographic characteristics of pregnant women in West Gojjam Zone

Variables	Intervention group (***n***_**1**_ = 313)	Control group (***n***_**2**_ = 332)	***P***
	Frequency (%)	Frequency (%)	
**Number of clusters**	11	11	
**Age (years)**			
< 20	25(8.0)	16(4.8)	
20–24	53(16.9)	74(22.3)	
25–29	103(32.9)	87(26.2)	0.085
30–34	74(23.7)	84(25.3)	
> =35	58(18.5)	71(21.4)	
**Religion**			
Orthodox	311(99.4)	330(99.4)	
Muslim	2(0.6)	2(0.6)	0.953
**Educational status**			
No formal education	260(83.1)	262(78.9)	
Formal education	53(16.9)	70(21.1)	0.180
**Occupational status**			
Housewife	149(47.6)	183(55.1)	
Farmer	164(52.4)	149(44.9)	0.060
**Marital status**			
Married	308(98.4)	331(99.7)	
Unmarried/ Divorced	5(1.6)	1(0.3)	0.087
**Husband education (*n*** **= 308,*n*** **= 331)**			
No formal education	238(77.3)	244(73.7)	
Primary education	50(16.2)	63(19.0)	0.575
Secondary and above education	20(6.5)	24(7.3)	
**Wealth index**			
Poorest	56(17.9)	60(18.0)	
Poor	72(23.0)	67(20.2)	
Medium	65(20.8)	62(18.7)	0.485
Rich	56(17.9)	78(23.5)	
Richest	64(20.4)	65(19.6)	
**Family Size**			
< 5	215(68.7)	240(72.3)	
**> =5**	98(31.3)	92(27.7)	0.316

*IG* intervention group, *CG* control group

### The health belief model and the theory of planned behavior constructs and their correlation with knowledge, dietary practice and nutritional status of pregnant women

There was a significant (*P* = < 0.001) improvement in the scores of the health belief model and the theory of planned behavior constructs among the intervention group. Whereas, in the control group detectable reduction was observed between the endline and baseline HBM and TPB constructs (Table 2). As shown in Table 3, except for the perceived barrier, all the other health belief model and theory of planned behavior constructs had a significant positive correlation with the nutritional status of pregnant women (*P < 0.001).*

**Table 2:** Comparison of the health belief model and the theory of planned behavior constructs score within and between the intervention and control groups among pregnant women in West Gojjam Zone, Ethiopia

HBM constructs	Study period	HBM & TPB constructs score		***P***
		Intervention group	Control group	
Perceived susceptibility	Baseline	3.6(±1.9)	3.9(±1.7)	0.051
	Endline	4.9(±1.7)	3.2(±1.8)	< 0.001
	P	< 0.001	< 0.001	
Perceived severity	Baseline	3.9(±1.5)	4.1(±1.4)	0.097
	Endline	4.2(±1.3)	3.4(±1.5)	< 0.001
	P	0.007	< 0.001	
Perceived benefits	Baseline	2.9(±1.7)	3.1(±1.7)	0.283
	Endline	4.3(±1.2)	2.8(±1.6)	< 0.001
	P	< 0.001	< 0.005	
Perceived barriers	Baseline	1.1(±1.1)	0.98 (±1.1)	0.127
	Endline	1.3(±1.1)	1.2(±1.0)	0.131
	P	0.026	0.014	
Intention	Baseline	19.4(±4.1)	19.7(±3.7)	0.200
	Endline	22.2 (±3.2)	18.8(±4.1)	< 0.001
	P	< 0.001	< 0.001	
Attitude	Baseline	62.7(±8.5)	63.8(±7.7)	0.074
	Endline	71.3 (±6.1)	61.4(±8.2)	< 0.001
	P	< 0.001	< 0.001	
Behavioral control	Baseline	8.8(±2.5)	8.7(±2.4)	0.850
	Endline	10.6(±2.4)	8.4(±2.6)	< 0.001
	P	< 0.001	0.015	
Subjective norms	Baseline	8.9(±2.8)	9.1 (±2.8)	0.391
	Endline	10.1 (±2.7)	8.4(±2.7)	< 0.001
	P	< 0.001	< 0.001	

*HBM* Health Belief Model, *TPB* Theory of planned behavior, *P* P-value

**Table 3:** Correlation of the health belief model and the theory of planned behavior constructs with knowledge, dietary practice and MUAC of pregnant women in West Gojjam Zones

	Intervention	Behavioral control	Subjective norms	Perceived severity	Perceived benefit	perceived barrier	Perceived susceptibility	Intention	Attitude	Knowledge	Dietary practice	MUAC
Intervention	1											
Behavioral control	.389^**^	1										
	.000											
Subjective norms	.312^**^	.700^**^	1									
	.000	.000										
Perceived severity	.274^**^	.480^**^	.442^**^	1								
	.000	.000	.000									
Perceived benefit	.470^**^	.565^**^	.542^**^	.611^**^	1							
	.000	.000	.000	.000								
Perceived barrier	.059	.003	.006	.050	.063	1						
	.129	.937	.879	.205	.109							
Perceived susceptibility	.440^**^	.601^**^	.568^**^	.682^**^	.822^**^	.039	1					
	.000	.000	.000	.000	.000	.324						
Intention	.421^**^	.711^**^	.644^**^	.526^**^	.629^**^	−.014	.658^**^	1				
	.000	.000	.000	.000	.000	.722	.000					
Attitude	.543^**^	.586^**^	.532^**^	.467^**^	.672^**^	−.032	.672^**^	.667^**^	1			
	.000	.000	.000	.000	.000	.416	.000	.000				
Knowledge	.522^**^	.458^**^	.466^**^	.412^**^	.572^**^	.022	.524^**^	.558^**^	.679^**^	1		
	.000	.000	.000	.000	.000	.582	.000	.000	.000			
Dietary practice	.307^**^	.296^**^	.272^**^	.221^**^	.263^**^	−.073	.312^**^	.314^**^	.328^**^	.397^**^	1	
	.000	.000	.000	.000	.000	.060	.000	.000	.000	.000		
MUAC	.168^**^	.119^**^	.144^**^	.130^**^	.167^**^	.037	.213^**^	.137^**^	.193^**^	.273^**^	.198^**^	1
	.000	.002	.000	.001	.000	.345	.000	.000	.000	.000	.000	

**Correlation is significant at the 0.01 level (2-tailed)

### Nutritional status of pregnant women

At the baseline, there was no statistically significant difference in the mean MUAC (23.13 ± 0.11 Vs 23.28 ± 0.08, *P* = 0.30) and the prevalence of undernutrition (43.8% Vs 39.8%, *P* = 0.28) between the two groups. After the implementation of the trial, the mean MUAC in the intervention arm has increased by 33% from the baseline while the prevalence of undernutrition was 16.7% lower in the intervention group compared with the control arm (30.6% Vs 47.3%, P = < 0.001). T-test results showed that the intervention improves the mean MUAC by 61% (Table 4).

**Table 4:** Differences between baseline and endline measurements of MUAC and difference of the differences between the intervention and control groups

	Intervention			Control				
	Baseline	Endline	Difference (EL-BL)	Baseline	Endline	Difference (EL-BL)	Difference of difference	
Variable	Mean(±SD)	Mean(±SD)	Mean(±SD)	Mean(±SD)	Mean(±SD)	Mean(±SD)	Mean(±SE)	*P*
MUAC	23.12(±2.0)	23.47(±1.6)	0.36(±1.1)	23.28(±1.8)	23.02(±1.9)	−0.25(±1.2)	0.61(±0.9)	< 0.001

*BL* Baseline*, EL* Endline*, SD* standard deviation*, SE* standard error

### Effect of the intervention on the nutritional status of pregnant women

The variance of the individual-level residual errors was 2.77 (variability of the average MUAC across individuals was 2.77), which was statistically significant (*p* = < 0.001). The intra-individual correlation coefficient was 0.79; this indicated the importance of accounting individual level time-invariant variables (fitting two-level models) (Table 5).

**Table 5:** Linear mixed predicting MUAC of pregnant women in West Gojjam Zone

Fixed effect	Model 1		Model 2		Model 3	
Variables	Estimate (SE)	95% CI	Estimate (SE)	95% CI	Estimate(SE)	95% CI
Intercept	23.225 (0.069)	(23.08,23.36)	23.259 (0.127)	(23.01,23.51)	22.781 (0.399)	(21.99,23.56)
Baseline MUAC (IG)			−0.163 (0.153)	(−0.46,0.13)	−0.116 (0.153)	(−0.41,0.18)
Endline MUAC (CG)			−0.255 (0.063)	(−0.37,-0.13)	**−0.**255 (0.063)	(−0.37,-0.13)
The intervention effect			**0.615 (0.091)**	(0.43,0.79)	**0.615 (0.091)**	(0.43,0.79)
Food secure			0.032 (0.086)	(−0.13, 0.19)	0.027 (0.085)	(−0.12, 0.17)
Age					0.011 (0.017)	(−0.02,0.04)
Family size					−0.013 (0.055)	(−0.12,0.09)
Have formal education					0.157 (0.191)	(−0.22,0.53)
Use latrine					0.322 (0.137)	(0.05,0.59)
Drink protected water					0.065 (0.148)	(−0.22,0.35)
**Random effect**						
Level two variance	2.77(0.17)		1.69(0.17)		1.28 (0.69)	
ICC	**0.798**		**0.442**		**0.564**	
AIC	**4623.28**		**4567.33**		**4573.13**	
Number of parameters	**3**		**9**		**19**	

*SE* Standard error, *CI* Confidence interval, *IG* Intervention group, *CG* Control group

After controlling for age, family size, food security, education, latrine utilization and source of drinking water women in the intervention group showed significant improvement in nutritional status at the end of the trial (β = 0.615, *p* = < 0.001). Whereas, MUAC of the pregnant women in the control group decreased by 25% on average (β = − 0.256, *p* = < 0.001) (Table 5).

## Discussion

This trial aimed to compare the effect of guided counseling using the health belief model and theory of planned behavior constructs to nutrition education given by the health system. The socio-demographic characteristics and nutritional status of pregnant women were similar at the baseline.

Nutrition education interventions given using behavioral models and theories were effective in improving nutrition habit [40–42]. In line with this fact, there was a significant improvement in the scores of the HBM and TPB constructs and dietary practices of the respondents in the intervention group compared with the baseline score and control group. Similarly, previous studies showed the positive significant effect of using HBM and TPB constructs during counseling to promote healthy nutritional behavior during pregnancy [43, 44].

Except for the perceived barrier, all the other HBM and TPB constructs had a significant positive correlation with the nutritional status of pregnant women*.* This is consistent to the study findings that reported the successful effect of nutrition education using the HBM and TPB constructs to bring behavioral change towards taking healthy diet [44, 45]. This might be due to the reason that women who attend nutrition education using the HBM perceived that the consequences of malnutrition are very severe and also they considered themselves as susceptible for the consequences of malnutrition. Besides, the women perceived that the benefits of taking adequate and diversified food outweigh the barriers of getting it. Their perception, in turn, can increases their attitude and behavioral control. These constructs further play a significant role in improving women’s intention of taking a balanced diet that directly contributes for increasing MUAC of the pregnant women.

Despite increasing nutrient requirement during pregnancy [46], dietary practices of Ethiopian pregnant women were similar with their practices before pregnancy [47]. As a result, significant numbers of pregnant women had undernutrition and the magnitude of malnutrition increased with increased gestational age [7, 48].

In line with the previous study findings in Ethiopia, the magnitude of undernutrition among pregnant women in the control group increased compared with the baseline prevalence. Whereas, in the intervention arm, the prevalence of undernutrition decreased by 13.2% than the baseline and it was 16.7% lower than the control group (30.6% Vs 47.3%, *P* = < 0.001). This is consistent with previous studies that reported positive effects of nutrition interventions in improving weight gain and nutritional status during pregnancy [15, 49].

After adjusting for age, family size, food security, education, source of drinking water and latrine utilization, pregnant women in the intervention group showed a significant improvement in nutritional status at the end of the trial (β = 0.615, *p* = < 0.001). Whereas, MUAC of the pregnant women in the control group decreased by 25% on average (β = −0.256, *p* = < 0.001). A study done in Tokyo also reported a positive effect of nutrition education intervention in improving the nutritional status of pregnant women [50].

The findings of this study showed that guided counseling using the HBM and TPB constructs was successful to improve women’s nutritional status. Unlike this intervention, nutrition education given by the health system was not effective in improving nutritional status of the pregnant women. This discrepancy is due to the difference in counseling methods. Because, this intervention used counseling guide, behavioral models and followed trimester based counseling. However, education given by the health care system is not using counseling guide, behavioral models and did not follow trimester based counseling. Therefore, these results suggest the need to amend the way nutrition education is given during pregnancy to bring about a positive behavior change.

This study acknowledges the following limitations during interpreting the results. Although intervention should start before conception to prevent negative consequence of malnutrition, It was implemented after conception. Besides, post intervention result may not have lasted longer since it was a relatively short term intervention.

## Conclusion

This study demonstrated that guided counseling using the HBM and the TPB was effective in improving nutritional status of pregnant women. Counseling using the HBM and the TPB is low cost and suitable intervention to improve knowledge, dietary practices and nutritional status of pregnant women. Thus, it is recommended to include the HBM and the TPB constructs to nutrition counseling guidelines. The findings also suggest developing nutrition guideline with core contents on diet during pregnancy.

## Supplementary information

**Additional file 1.** CONSORT 2010 checklist of information to include when reporting a randomised trial*.

**Additional file 2.** Study clusters in the study area.

**Additional file 3.** Theory of planned behavior and health belief model constructs.

## Data Availability

All the data related to this research are available in the text, tables or figures.

## Abbreviations

AIC: Akaike Information Criterion
ANC: Antenatal Care
CG: Control Group
CONSORT: Consolidated Standards of Reporting Trials
EDHS: Ethiopian Demographic and Health Survey
HBM: Health Belief Model
ICC: Intra-class Correlation Coefficient
IG: Intervention Group
MUAC: Mid-upper Arm Circumference
PCA: Principal Component Analysis
SD: Standard Deviation
SE: Standard Error
TPB: Theory of Planned Behavior
