# Supplementary figures and images for: Effect of guided counseling on nutritional status of pregnant women in West Gojjam zone, Ethiopia: a cluster-randomized controlled trial
Source: Nutr J. 2020 Apr 28;19:38. doi: 10.1186/s12937-020-00536-w (PMC7189500; doi:10.1186/s12937-020-00536-w)

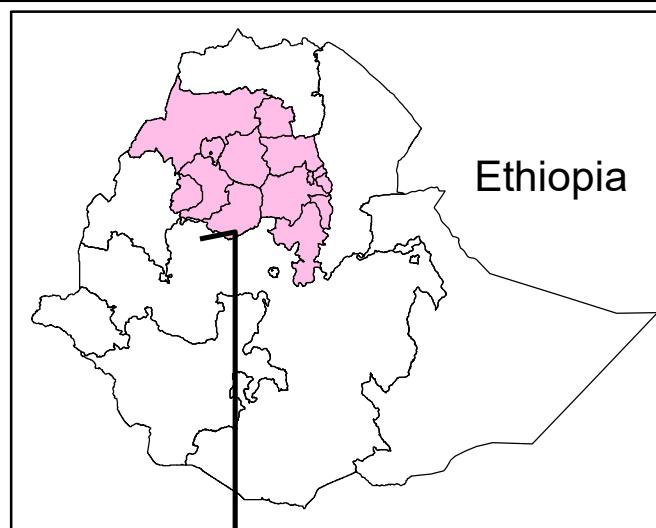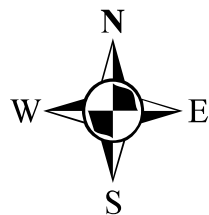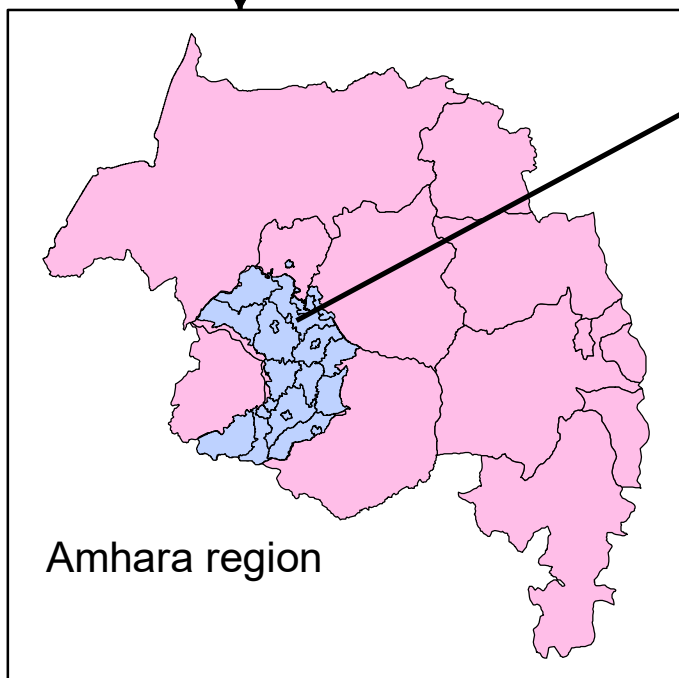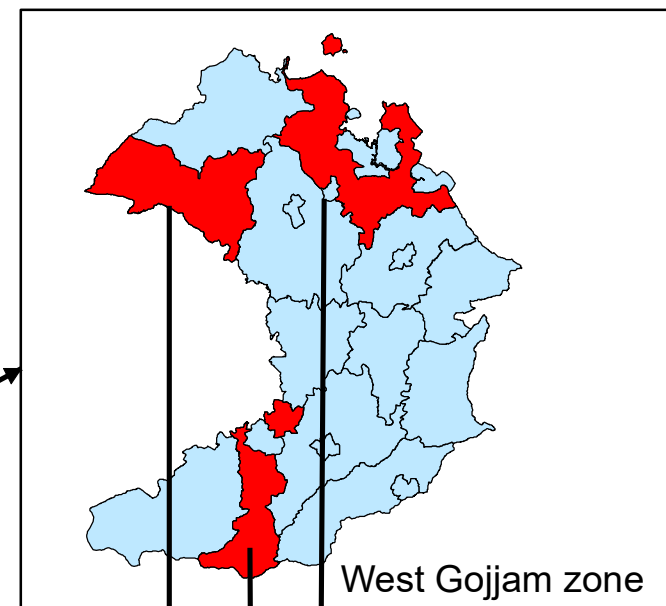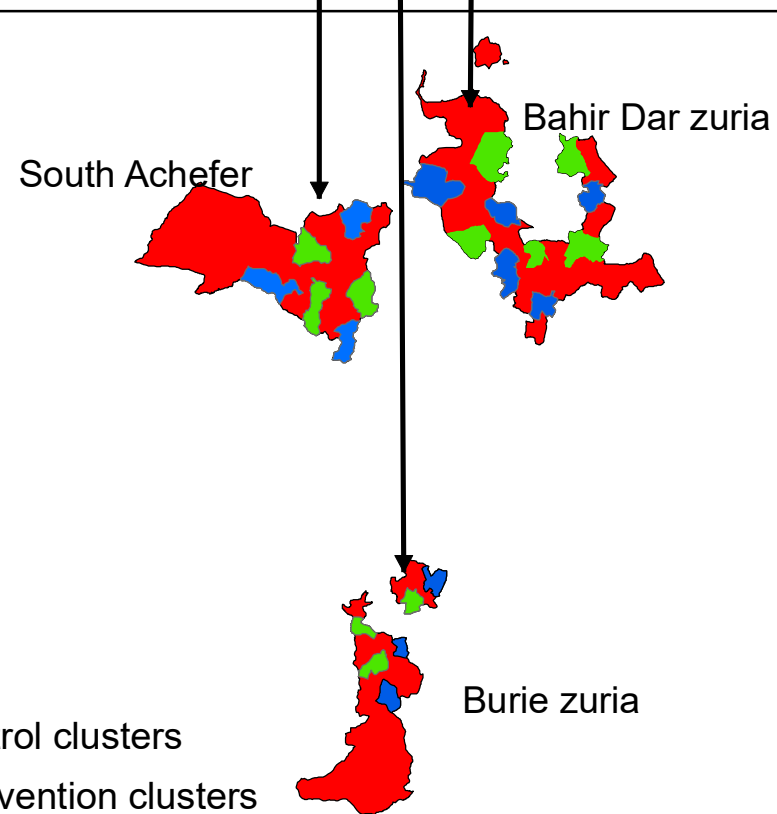

### Legend

- 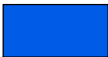 Control clusters
- 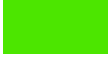 Intervention clusters

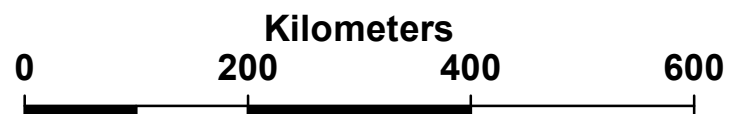

Supplement: Supplementary file 2 — Additional file 2. Study clusters in the study area. [file 12937_2020_536_MOESM2_ESM.pdf]
