# Supplementary material for: Effect of guided counseling on nutritional status of pregnant women in West Gojjam zone, Ethiopia: a cluster-randomized controlled trial
Source: Nutr J. 2020 Apr 28;19:38. doi: 10.1186/s12937-020-00536-w (PMC7189500; doi:10.1186/s12937-020-00536-w)
Supplement: Supplementary file 3 — Additional file 3. Theory of planned behavior and health belief model constructs. [file 12937_2020_536_MOESM3_ESM.docx]

**External factors**

Knowledge

Perceived susceptibility

Perceived severity

Perceived barriers

Attitude

Intention

Dietary practice

Health value

Subjective norms

**Cues to action**

-professionals, family members

Perceived behavioral control

**Background &perception** **belief & attitude intention behavior**

Additional file 3: A new model: combined Health Belief Model and Theory of Planned Behavior adopted from SUN X *et al[*[*1*](#_ENREF_1)*]*.

**Reference**

1. Sun X, Guo Y, Wang S, Sun J: **Predicting Iron-Fortified Soy Sauce Consumption Intention: Application of the Theory of Planned Behavior and Health Belief Model.** *J Nutr Educ Behav* 2006, **38:**276-285.
